# Patterns of new glucagon‐like peptide‐1 receptor agonist use in patients with type 2 diabetes during 2014–2019 from a US database: prescriber and patient characteristics

**DOI:** 10.1111/1753-0407.13363

**Published:** 2023-02-16

**Authors:** Ildiko Lingvay, Vanita R. Aroda, Julie Broe Honoré, Anne S. Ersbøll, Lise Lotte Nystrup Husemoen, Anders Boeck Jensen, Kasper Sommer Matthiessen, Mikhail Naum Kosiborod

**Affiliations:** ^1^ University of Texas Southwestern Medical Center Dallas Texas USA; ^2^ Brigham and Women's Hospital and Harvard Medical School Boston Massachusetts USA; ^3^ Novo Nordisk A/S Søborg Denmark; ^4^ Saint Luke's Mid America Heart Institute and University of Missouri‐Kansas City Kansas City Missouri USA

**Keywords:** ASCVD, cardiovascular, CVOT, GLP‐1 RA, type 2 diabetes

## Abstract

**Highlights** This study demonstrates that initiation of glucagon‐like peptide‐1 receptor agonists among individuals with type 2 diabetes (T2D), including those with concomitant atherosclerotic cardiovascular disease (ASCVD), has remained low in the United States between 2014 and 2019, despite clinical evidence supporting their use for cardiovascular risk reduction. These findings add to the existing literature to highlight a gap in adherence to current practice guidelines, which suggests that most patients with T2D and ASCVD in the United States may not be receiving optimal risk‐reducing therapies.

To the Editor

Multiple cardiovascular outcome trials (CVOTs) have demonstrated that sodium‐glucose cotransporter‐2 inhibitors (SGLT2is) and glucagon‐like peptide‐1 receptor agonists (GLP‐1 RAs) reduce cardiovascular (CV) risk in individuals with type 2 diabetes (T2D) and CV disease (CVD) or at high risk for CVD.^1^ Consequently, several agents within these classes are now recommended in both diabetes and CV international practice guidelines.^1^, ^2^, ^3^

However, a granular understanding of whether these recommendations have led to increases in new prescriptions among certain patient subgroups, or across clinician specialties, is lacking.^4^, ^5^ We aimed to investigate the incidence of new GLP‐1 RA use in patients with T2D, with or without atherosclerotic CVD (ASCVD), between 2014 and 2019, a period that encompassed key CVOT publications and an updated GLP‐1 RA label indication.

## 1 METHODS

This descriptive study used data from a United States (US) claims data repository (Optum® deidentified Clinformatics® Data Mart [CDM] Database 2004–2020). Consecutive cross‐sectional cohorts corresponding to each year during 2014–2019 were identified, comprising adults with T2D and no GLP‐1 RA use for ≥1 year prior to the study year of interest.

T2D was defined as a recorded diagnosis prior to the start of the study year of interest, with no recorded type 1 diabetes diagnosis. ASCVD was defined as a history of myocardial infarction, cerebral infarction, atherosclerosis, revascularization procedures, peripheral artery disease, chronic ischemic heart disease, or unstable angina (Table S1). Patients with a new GLP‐1 RA prescription filled during the study year of interest were identified by National Drug Codes (Table S2) and defined as new GLP‐1 RA users. The GLP‐1 RA prescribers' specialties were categorized as cardiology, endocrinology, family medicine/general practitioner, internal medicine, and other.

For each study year, incidence rates of new GLP‐1 RA use were calculated per 100 patient‐years for the overall T2D population, subpopulation of patients with T2D and ASCVD, and according to prescribing specialty.

Further details concerning the methodology are in the Supplementary Appendix S1.

## 2 RESULTS

A total of 2 935 736 eligible patients with T2D were identified, of whom 107 574 (3.7%) were new users of GLP‐1 RAs. Among 1 415 396 patients with T2D and concomitant ASCVD, 37515 (2.7%) were new GLP‐1 RA users.

New GLP‐1 RA users were younger than non‐GLP‐1 RA users (total group mean: 60.1 vs 68.3 years) and had higher mean glycated hemoglobin levels (8.6% vs 6.7%; Table 1). Prevalences of obesity and microvascular complications were higher, and of ASCVD, stroke, and angina pectoris were lower among new GLP‐1 RA users vs non‐GLP‐1 RA users (Table 1). Use of CV‐related medications was observed in a higher proportion of new GLP‐1 RA users than non‐GLP‐1 RA users (Table 1).

**TABLE 1:** Characteristics of patients with T2D with and without a new GLP‐1 RA prescription fill from the US Optum® database during 2014 to 2019, reported overall and annually

Characteristic	T2D population													
	New GLP‐1 RA users							Non‐GLP‐1 RA users						
	2014	2015	2016	2017	2018	2019	Total	2014	2015	2016	2017	2018	2019	Total
*N*	6471	8620	12 134	19 326	26 109	34 914	107 574	936 734	998 065	1 149 153	1 357 446	1 599 922	1 723 371	7 764 691
Mean age ± SD, years	56.8 ± 11.4	57.0 ± 11.2	58.1 ± 11.5	59.8 ± 11.7	61.2 ± 11.8	61.7 ± 11.9*	60.1 ± 11.9	65.8 ± 14.1	66.6 ± 14.0	67.6 ± 13.7	68.4 ± 13.2	69.4 ± 12.9	69.9 ± 12.7	68.3 ± 13.4
Female, *n* (%)	3326 (51.4)	4509 (52.3)	6436 (53.0)	10 112 (52.3)	13 573 (52.0)	18 160 (52.0)	56 116 (52.2)	478 503 (51.1)	513 867 (51.5)	595 456 (51.8)	711 437 (52.4)	837 460 (52.3)	902 860 (52.4)	4 039 583 (52.0)
Mean HbA_1c_ ± SD, %	8.7 ± 1.8	8.6 ± 1.9	8.5 ± 1.8	8.6 ± 1.8	8.7 ± 1.8	8.7 ± 1.9	8.6 ± 1.8	6.7 ± 1.3	6.8 ± 1.4	6.7 ± 1.4	6.8 ± 1.4	6.6 ± 1.3	6.7 ± 1.3	6.7 ± 1.4
Mean eGFR ± SD, mL/min/1.73 m^2^	86.9 ± 22.3	84.3 ± 22.7	84.6 ± 23.2	81.4 ± 24.0	79.1 ± 24.0	78.7 ± 24.2*	80.9 ± 23.9	78.4 ± 22.9	76.8 ± 23.0	75.5 ± 22.9	75.3 ± 22.9	73.8 ± 22.4	72.8 ± 22.1	74.8 ± 22.7
Albuminuria*														
Macroalbuminuria, *n* (%)	21 (4.9)	41 (6.1)	70 (6.7)	155 (9.0)	224 (9.3)	294 (9.8)	804 (8.7)	403 (5.2)	1298 (5.8)	1547 (5.7)	2160 (6.2)	3178 (6.4)	3873 (6.3)	12 435 (6.2)
Microalbuminuria, *n* (%)	106 (24.6)	209 (31.3)	355 (33.8)	539 (31.2)	801 (33.3)	1015 (33.9)	3025 (32.6)	1803 (23.2)	5435 (24.4)	6785 (25.2)	9144 (26.2)	13 159 (26.5)	16 232 (26.5)	52 420 (25.9)
Lipid profile, mean ± SD														
Total cholesterol (mg/dL)	177.1 ± 44.3	176.9 ± 45.7	177.8 ± 44.0	175.3 ± 45.2	172.7 ± 46.0	170.8 ± 46.8*	173.8 ± 45.8	179.1 ± 41.4	178.4 ± 41.9	177.3 ± 42.3	176.5 ± 42.8	173.9 ± 42.3	169.8 ± 41.6	174.8 ± 42.2
Triglycerides (mg/dL)	210.2 ± 188.7	206.9 ± 178.5	210.8 ± 195	205.8 ± 177.7	205.0 ± 193.5	200.2 ± 191.0*	204.8 ± 188.6	150.1 ± 109.1	151.8 ± 115.9	152.9 ± 119	152.7 ± 117.2	151.7 ± 111.0	147.1 ± 106.3	150.7 ± 112.3
LDL cholesterol (mg/dL)	93.7 ± 34.9	93.0 ± 36.3	92.7 ± 34.3	91.2 ± 35.9	90.4 ± 35.6	89.7 ± 36.0*	91.0 ± 35.7	98.6 ± 34.4	97.7 ± 34.8	96.0 ± 35.0	95.5 ± 35.2	94.1 ± 34.7	91.3 ± 34.3	94.7 ± 34.8
HDL cholesterol (mg/dL)	44.5 ± 12.6	44.4 ± 13.2	45.0 ± 13.0	45.2 ± 13.5	44.8 ± 12.7	45.0 ± 12.6	44.9 ± 12.9	50.9 ± 15.5	50.8 ± 15.8	50.9 ± 15.9	50.9 ± 16.1	50.9 ± 15.6	50.6 ± 15.3	50.8 ± 15.7
GLDs														
Mean number of GLDs*														
1 GLD, *n* (%)	1510 (23.3)	1901 (22.1)	2697 (22.2)	4270 (22.1)	5798 (22.2)	8038 (23.0)	24 214 (22.5)	220 273 (23.5)	240 010 (24.0)	278 758 (24.3)	356 988 (26.3)	426 846 (26.7)	457 637 (26.6)	1 980 512 (25.5)
2 GLDs, *n* (%)	1947 (30.1)	2347 (27.2)	3491 (28.8)	5654 (29.3)	7951 (30.5)	10 624 (30.4)	32 014 (29.8)	120 606 (12.9)	126 930 (12.7)	144 098 (12.5)	186 341 (13.7)	220 304 (13.8)	233 304 (13.5)	1 031 583 (13.3)
3 GLDs, *n* (%)	1445 (22.3)	1920 (22.3)	2769 (22.8)	4612 (23.9)	6287 (24.1)	8241 (23.6)	25 274 (23.5)	42 649 (4.6)	46 732 (4.7)	53 405 (4.6)	70 195 (5.2)	85 690 (5.4)	91 782 (5.3)	390 453 (5.0)
4+ GLDs, *n* (%)	1080 (16.7)	1689 (19.6)	2151 (17.7)	3289 (17.0)	4294 (16.4)	5431 (15.6)	17 934 (16.7)	9606 (1.0)	12 318 (1.2)	15 112 (1.3)	19 866 (1.5)	24 670 (1.5)	27 124 (1.6)	108 696 (1.4)
Biguanide, *n* (%)	4981 (77.0)	6466 (75.0)	9141 (75.3)	14 222 (73.6)	19 317 (74.0)	25 487 (73.0)*	79 614 (74.0)	315 403 (33.7)	346 658 (34.7)	400 943 (35.0)	511 995 (37.7)	614 813 (38.4)	656 315 (38.1)	2 846 127 (36.7)
Sulfonylurea, *n* (%)	2912 (45.0)	3447 (40.0)	4877 (40.2)	7705 (40.0)	10 589 (40.6)	13 421 (38.4)*	42 951 (39.9)	150 794 (16.1)	153 959 (15.4)	170 994 (14.9)	217 300 (16.0)	253 239 (15.8)	263 196 (15.3)	1 209 482 (15.6)
SGLT2i, *n* (%)	409 (6.3)	1472 (17.1)	2383 (19.6)	3913 (20.2)	5305 (20.3)	7273 (20.8)*	20 755 (19.3)	2364 (0.3)	11 738 (1.2)	24 611 (2.1)	34 522 (2.5)	47 128 (2.9)	53 705 (3.1)	174 068 (2.2)
Insulin, *n* (%)	1704 (26.3)	2488 (28.9)	3323 (27.4)	6036 (31.2)	8562 (32.8)	11 483 (32.9)*	33 596 (31.2)	56 834 (6.1)	64 178 (6.4)	73 960 (6.4)	105 182 (7.7)	125 715 (7.9)	138 250 (8.0)	564 119 (7.3)
CV‐related medication, *n* (%)														
Antihypertensive drugs	5160 (79.7)	6869 (80.0)	9798 (80.7)	15 938 (82.5)	21 943 (84.0)	29 270 (83.8)	88 978 (82.7)	619 613 (66.1)	658 592 (66.0)	752 184 (65.5)	935 430 (68.9)	1 104 590 (69.0)	1 191 909 (69.2)	5 262 318 (67.6)
Lipid‐lowering drugs	4531 (70.0)	5987 (69.5)	8578 (70.7)	14 052 (72.7)	19 519 (74.8)	26 567 (76.1)	79 234 (73.7)	490 538 (52.4)	523 843 (52.5)	598 731 (52.1)	751 850 (55.4)	899 639 (56.2)	984 329 (57.1)	4 248 930 (54.7)
Mean Charlson comorbidity index ± SD, score	2.4 ± 2.4	2.7 ± 2.3	3.2 ± 2.2	3.3 ± 2.2	3.5 ± 2.3	3.6 ± 2.3*	3.3 ± 2.3	2.7 ± 2.7	2.8 ± 2.7	3.2 ± 2.7	3.5 ± 2.6	3.6 ± 2.6	3.8 ± 2.7	3.3 ± 2.7
Comorbidities, %														
CV event^a^	17.1	17.1	21.7	26.4	28.7	30.0*	26.3	24.5	25.2	29.0	32.5	34.0	35.4	31.0
ASCVD	32.5	32.3	33.3	36.3	38.9	40.3*	37.3	42.3	43.1	44.3	44.8	45.9	47.7	45.1
Heart failure	8.5	9.4	12.3	16.0	19.3	20.8*	17.0	13.7	14.0	16.2	19.7	22.0	24.1	19.2
Stroke	10.9	10.2	10.3	10.2	10.3	10.4	10.4	17.4	17.9	18.3	16.8	15.8	15.7	16.8
MI	3.3	3.5	4.8	7.1	8.5	9.2*	7.4	4.7	4.8	5.7	7.7	8.9	9.9	7.4
Angina pectoris	10.5	10.6	11.1	9.9	9.5	8.9	9.7	15.4	16.0	16.6	14.7	12.9	12.1	14.3
Hypertension	89.1	89.0	89.5	90.3	90.4	90.5*	90.1	84.5	84.8	85.7	87.0	87.7	88.4	86.7
Coronary arterial revascularization	2.8	2.5	2.6	2.1	2.0	1.9	2.0	3.0	2.9	2.9	2.5	2.2	2.1	2.5
Peripheral artery disease	10.5	11.0	14.8	19.1	23.2	25.8*	20.6	15.4	16.0	18.5	22.0	24.4	27.2	21.6
Obesity	57.1	60.1	64.7	67.4	69.5	72.0*	67.9	29.1	31.4	34.9	39.2	43.2	47.6	39.0
Retinopathy	15.5	16.6	21.1	25.0	27.8	30.3*	25.7	9.9	10.3	14.2	20.8	24.6	28.0	19.5
Neuropathy	39.3	42.4	43.9	43.1	42.7	42.7*	42.7	33.4	35.5	37.2	34.5	32.7	32.3	34.0
History of CKD	20.3	22.3	25.6	29.0	31.7	33.6*	29.7	22.1	23.9	26.0	28.0	29.7	31.3	27.6

Abbreviations: ASCVD, atherosclerotic cardiovascular disease; CKD, chronic kidney disease; CV, cardiovascular; eGFR, estimated glomerular filtration rate; GLD, glucose‐lowering drug; GLP‐1 RA, glucagon‐like peptide‐1 receptor agonist; HbA_1c_, glycated hemoglobin; HDL, high‐density lipoprotein; LDL, low‐density lipoprotein; MI, myocardial infarction; *N*, total number of participants; *n*, number of participants; SGLT2i, sodium‐glucose co‐transporter‐2 inhibitor; T2D, type 2 diabetes.

* *P* < .05 indicates a statistically significant difference in value or proportion comparing 2014 and 2019 in new GLP‐1 RA users relative to non‐GLP‐1 RA users. This was tested using a linear model for continuous data and a Poisson regression model for categorical data.

^a^ CV event comprises cases of MI and stroke.

**FIGURE 1:**
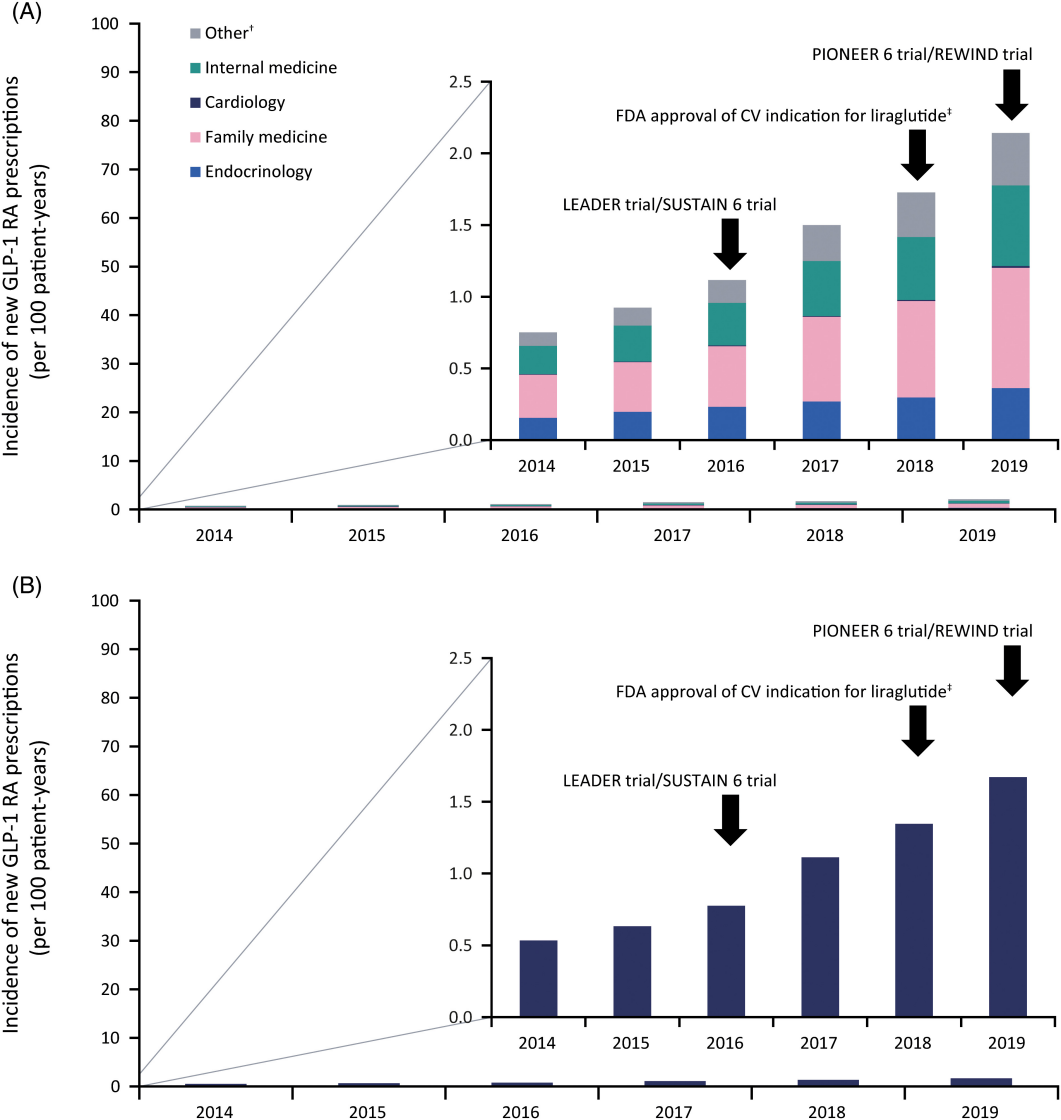
Incidence of new GLP‐1 RA prescription fills during 2014–2019 in (A) patients with T2D according to specialty, and (B) the subgroup of patients with T2D and ASCVD. Black arrows demarcate publication of landmark CVOTs and updates to GLP‐1 RA label indication. Details of the CVOT trials have been published previously.^4^, ^5^, ^13^, ^14^ ^†^Other: all other specialties that prescribed GLP‐1 RA, as reported in the US Optum® database. ^‡^Within patients with T2D. ASCVD, atherosclerotic cardiovascular disease; CV, cardiovascular; CVOT, cardiovascular outcome trial; FDA, Food and Drug Administration; GLP‐1 RA, glucagon‐like peptide‐1 receptor agonist; LEADER, Liraglutide Effect and Action in Diabetes: Evaluation of Cardiovascular Outcome Results; PIONEER 6, Peptide Innovation for Early Diabetes Treatment; REWIND, Researching Cardiovascular Events With a Weekly Incretin in Diabetes; SUSTAIN 6, Trial to Evaluate Cardiovascular and Other Long‐term Outcomes with Semaglutide in Subjects with Type 2 Diabetes; T2D, type 2 diabetes.

Overall incidence of new GLP‐1 RA use remained low throughout the study period, although it increased modestly from 0.75 to 2.14 per 100 patient‐years between 2014 and 2019 (Figure 1A). Family medicine specialty was the greatest contributor to the prescribing incidence (≈40% of prescriptions each year). Endocrinology accounted for approx. 19%, and cardiology contributed the least (<1% of prescriptions). Likewise, incidence of new GLP‐1 RA use among the patient subpopulation with T2D and ASCVD was low throughout the study and demonstrated a modest increase (from 0.53 to 1.67 per 100 patient‐years between 2014 and 2019; Figure 1B).

## 3 COMMENT

This study, which, to our knowledge, examined the largest population of patients with T2D and ASCVD to date, found that, despite increasing modestly, initiation of GLP‐1 RAs remained low in the US between 2014 and 2019, particularly among individuals with concomitant ASCVD. Analysis showed that cardiologists contributed to <1% of new prescriptions. Furthermore, the proportion of patients with ASCVD was lower among patients who had a filled GLP‐1 RA prescription compared to those without. This does not align with current practice guidelines and may represent a delay in translation of the large CVOTs into routine clinical practice.

Our findings are in line with a recent study showing that monthly prescribing of GLP‐1 RAs and SGLT2is by US cardiologists remained very low following the publication of CVOT data.^6^ Similarly, population‐based data to date show little impact of the publication of the key CVOTs on GLP‐1 RA prescribing rates.^7^, ^8^, ^9^, ^10^, ^11^, ^12^

There are several limitations to the current study, including the use of filled prescription data to denote medication use and shifts in insurance coverage in the US claims repository during the study that could have affected access to branded medications. Recommendations for GLP‐1 RA use in patients with T2D and CVD were not incorporated into international guidelines until late 2018; therefore, their potential impact on new GLP‐1 RA prescriptions could not be fully captured in our study, which concluded in 2019.^2^

In summary, we observed that use of GLP‐1 RAs among individuals with T2D, including those with ASCVD, remained very low between 2014 and 2019, despite availability of clinical evidence supporting their use for CV risk reduction. Our study suggests that GLP‐1 RAs continue to be perceived predominantly as glucose‐lowering medications and indicates that most patients with T2D and ASCVD in the US may not be receiving optimal risk‐reducing therapies.

## AUTHOR CONTRIBUTIONS

We confirm that all authors have contributed significantly and, in keeping with the latest guidelines of the International Committee of Medical Journal Editors, each author's contributions are as follows: Ildiko Lingvay, Vanita R. Aroda, Julie Broe Honore, Anne S. Ersbøll, Lise Lotte Nystrup Husemoen, Anders Boeck Jensen, Kasper Sommer Matthiessen, and Mikhail Naum Kosiborod provided substantial contribution to the concept and design of the work, interpretation of the data, preparation of the first draft of the manuscript, and critical revision of the work for important intellectual content. Furthermore, Anders Boeck Jensen was responsible for acquisition and analysis of the data. All authors critically revised the drafts and approved the final version to be published.

## DISCLOSURE

Ildiko Lingvay has received research grants from Merck, Mylan, Novo Nordisk, Pfizer, and Sanofi, as well as personal fees from AstraZeneca, Bayer, Boehringer Ingelheim, Duke CRI, Intarcia, Intercept, Janssen, Lilly, Mannkind, Novo Nordisk, Sanofi, TARGET Pharma, Valeritas, and Zealand Pharma, and nonfinancial support from AstraZeneca, Boehringer Ingelheim, Janssen, Lilly, Merck, Novo Nordisk, Pfizer, and Sanofi. Vanita R. Aroda has received consultancy fees from Applied Therapeutics, Fractyl, Novo Nordisk, Pfizer, and Sanofi; research support via institutional contracts from Applied Therapeutics/Medpace, Eli Lilly, Premier/Fractyl, Novo Nordisk, and Sanofi/Medpace; and her spouse is an employee at Janssen. Mikhail Naum Kosiborod has received research grants from AstraZeneca and Boehringer Ingelheim; consultancy fees from Alnylam, Amgen, Applied Therapeutics, AstraZeneca, Bayer, Boehringer Ingelheim, Cytokinetics, Dexcom, Eli Lilly, Esperion Therapeutics, Janssen, Lexicon, Merck (Diabetes and Cardiovascular), Novo Nordisk, Pharmacosmos, Pfizer, Sanofi, and Vifor Pharma; and other research support from AstraZeneca. Julie Broe Honore, Anne S. Ersbøll, Lise Lotte Nystrup Husemoen, Anders Boeck Jensen, and Kasper Sommer Matthiessen are employees of Novo Nordisk A/S, a producer of GLP‐1 RAs and other antidiabetic drugs. Lise Lotte Nystrup Husemoen is also a Novo Nordisk A/S shareholder.

## ETHICS STATEMENT

Patient consent and ethics approval were not required for the use of these anonymized prescription data. The authors had permission to access the data from the Optum® deidentified CDM Database (2004–2020), granted by Optum®.

## Supporting information


**Appendix S1.** Methodology.
**Table S1.** Diagnoses' codes and descriptions used to identify patient characteristics from the Optum® database.
**Table S2.** National Drug Codes used to identify GLP‐1 RA users from the Optum® database.
