# Supplementary material for: Patterns of new glucagon‐like peptide‐1 receptor agonist use in patients with type 2 diabetes during 2014–2019 from a US database: prescriber and patient characteristics
Source: J Diabetes. 2023 Feb 16;15(2):190–5. doi: 10.1111/1753-0407.13363 (PMC9934953; doi:10.1111/1753-0407.13363)
Supplement: Supplementary file 1 — Appendix S1. Methodology. Table S1. Diagnoses' codes and descriptions used to identify patient characteristics from the Optum® database. Table S2. National Drug Codes used to identify GLP‐1 RA users from the Optum® database. [file JDB-15-190-s001.pdf]

SUPPORTING INFORMATION

Title: Patterns of new glucagon-like peptide-1 receptor agonist use in patients with type 2 diabetes during 2014–2019 from a US database: prescriber and patient characteristics

Authors: Ildiko Lingvay, Vanita R. Aroda, Julie Broe Honore, Anne Ersbøll, Lise Lotte Nystrup Husemoen, Anders B. Jensen, Kasper Sommer Matthiessen, Mikhail Naum Kosiborod

Appendix S1. Methodology

This is a descriptive study of retrospective data from Optum® de-identified Clinformatics® Data Mart (CDM) Database (2004–2020), a claims database based on filled prescriptions for people enrolled in large commercial and Medicare Advantage health plans in the US. CDM administrative claims are submitted for all medical and pharmacy healthcare services with information related to healthcare costs and resource utilization for payment by providers and pharmacies. Submitted claims are verified, adjudicated, and de-identified by a third-party vendor prior to inclusion.

Data from Optum® were used to extract consecutive cross-sectional cohorts corresponding to each year during 2014–2019. Eligible individuals were aged ≥18 years, had type 2 diabetes (T2D), and no filled prescription of glucagon-like peptide-1 receptor agonist (GLP-1 RA) for at least 1 year prior to the study year of interest. They had active insurance enrolment for at least 1 year before the study year of interest and at least one claim (visit, prescription, procedure, laboratory test, or diagnosis) recorded in the database during that year. Diagnoses and procedures were coded in the International Classification of Disease 9th/10th revision, clinical modification (9CM/10CM) (**Table S1**). T2D was defined as a recorded T2D diagnosis prior to the start of the study year of interest. Patients with a recorded diagnosis of both type 1 diabetes and T2D were excluded from the cohort. A subset of patients with T2D and atherosclerotic cardiovascular disease (ASCVD) was also identified. ASCVD was defined as a history of myocardial infarction (MI), cerebral infarction, atherosclerosis, revascularization procedures, peripheral artery disease, chronic ischemic heart disease, or unstable angina (**Table S1**).

Patients with a new GLP-1 RA prescription filled during the study year of interest were identified by National Drug Codes (**Table S2**) and defined as new GLP-1 RA users. Patients could be included in more than one study cohort if no GLP-1 RA prescriptions were filled for >1 year. The population of non-GLP-1 RA users was defined as those who did not have a GLP-1 RA prescription filled during the study period and was used as a comparison population within these analyses.

For all new GLP-1 RA users, the speciality of the prescriber was determined from the provider category in Optum®. This category is based on provider speciality as identified in the contract with Optum®'s affiliate payer. The specialties were categorized as follows: cardiology (including cardiologists, cardiac, thoracic and vascular surgeons, and cardiac nurse practitioners), endocrinology, family medicine/general practitioner, internal medicine and other (speciality not in a particular scope or unknown).

Data were obtained on patient age, gender, and laboratory assessments, including glycated hemoglobin, estimated glomerular filtration rate (eGFR), urine albumin, and lipid profile, within 6

months prior to the first GLP-1 RA prescription fill date (or start of the study year for non-GLP-1 RA users). eGFR was calculated based on the "Chronic Kidney Disease Epidemiology Collaboration (CKD-EPI)" formula.[1] Data were also obtained on previous diagnoses including heart failure, stroke, MI, angina pectoris, hypertension, coronary arterial revascularization, peripheral artery disease, microvascular complications, and obesity. MI and stroke were pooled into a "cardiovascular (CV) event" category. Diagnoses were included if a patient had these recorded on or prior to their first GLP-1 RA prescription fill (or start of the study year for non-GLP-1 RA users). Use of antidiabetes medications (type and number of different types) and CV disease-related medications (including anti-hypertensive and lipid-lowering drugs) within 1 year prior to a new GLP-1 RA prescription fill (or start of study year for non-GLP-1 RA users) were evaluated. Disease severity and comorbidity burden were assessed using the Charlson comorbidity index (CCI).[2]

For each calendar year during 2014–2019, incidence rates of new GLP-1 RA use were calculated per 100 patient-years for the population of patients with T2D. Similar analyses were performed for the subpopulation of patients with both ASCVD and T2D, and among each prescribing speciality. Incidence rate was calculated as the number of individuals with at least one GLP-1 RA prescription filled during the year of interest, divided by the total person-years at risk (for all GLP-1 RA users and non-GLP-1 RA users).

Patient characteristics are provided as descriptive statistics (n [%] for categorical variables and mean ± standard deviation for continuous variables). Differences in mean values for age and laboratory assessments between 2014 and 2019 were compared using a simple linear model with year (2014 vs 2019), population (non-GLP-1 RA users vs GLP-1 RA users), and their interaction as variables. Significance of *P*-values for the interactions are reported ($P<0.05$). The interaction can be interpreted as the change from 2014 to 2019 among GLP-1 RA users that cannot be attributed to a change among the background population of non-GLP-1 RA users. Categorical variables were tested with Poisson regression models using the same variables as for the linear model and the significance of *P*-values for the interaction reported. CCI was made categorical by comparing CCI ≥3 to CCI <3. For glucose-lowering drugs (GLDs), the total number of GLDs for all patients was used for the Poisson model (instead of the patient count). Statistical testing was not carried out if there were fewer than five patients either with a measurement or a comorbidity diagnosis in either 2014 or 2019.

References

1. Levey AS, Stevens LA, Schmid CH, et al. A new equation to estimate glomerular filtration rate. *Ann Intern Med.* 2009;150:604-612.
2. Glasheen WP, Cordier T, Gumpina R, Haugh G, Davis J, Renda A. Charlson Comorbidity Index: ICD-9 Update and ICD-10 Translation. *Am Health Drug Benefits.* 2019;12:188-197.

Supplementary Table S1. Diagnoses' codes and descriptions used to identify patient characteristics from the Optum® database

Codelist name	ICD identifier	Code	Description
T1D	ICD10CM	E10	Type 1 diabetes mellitus
T1D	ICD10CM	E10.1	Type 1 diabetes mellitus with ketoacidosis
T1D	ICD10CM	E10.10	Type 1 diabetes mellitus with ketoacidosis without coma
T1D	ICD10CM	E10.11	Type 1 diabetes mellitus with ketoacidosis with coma
T1D	ICD10CM	E10.2	Type 1 diabetes mellitus with kidney complications
T1D	ICD10CM	E10.21	Type 1 diabetes mellitus with diabetic nephropathy
T1D	ICD10CM	E10.22	Type 1 diabetes mellitus with diabetic chronic kidney disease
T1D	ICD10CM	E10.29	Type 1 diabetes mellitus with other diabetic kidney complication
T1D	ICD10CM	E10.3	Type 1 diabetes mellitus with ophthalmic complications
T1D	ICD10CM	E10.31	Type 1 diabetes mellitus with unspecified diabetic retinopathy
T1D	ICD10CM	E10.311	Type 1 diabetes mellitus with unspecified diabetic retinopathy with macular edema
T1D	ICD10CM	E10.319	Type 1 diabetes mellitus with unspecified diabetic retinopathy without macular edema
T1D	ICD10CM	E10.32	Type 1 diabetes mellitus with mild nonproliferative diabetic retinopathy
T1D	ICD10CM	E10.321	Type 1 diabetes mellitus with mild nonproliferative diabetic retinopathy with macular edema
T1D	ICD10CM	E10.3211	Type 1 diabetes mellitus with mild nonproliferative diabetic retinopathy with macular edema, right eye
T1D	ICD10CM	E10.3212	Type 1 diabetes mellitus with mild nonproliferative diabetic retinopathy with macular edema, left eye
T1D	ICD10CM	E10.3213	Type 1 diabetes mellitus with mild nonproliferative diabetic retinopathy with macular edema, bilateral
T1D	ICD10CM	E10.3219	Type 1 diabetes mellitus with mild nonproliferative diabetic retinopathy with macular edema, unspecified eye
T1D	ICD10CM	E10.329	Type 1 diabetes mellitus with mild nonproliferative diabetic retinopathy without macular edema
T1D	ICD10CM	E10.3291	Type 1 diabetes mellitus with mild nonproliferative diabetic retinopathy without macular edema, right eye
T1D	ICD10CM	E10.3292	Type 1 diabetes mellitus with mild nonproliferative diabetic retinopathy without macular edema, left eye

T1D	ICD10CM	E10.3293	Type 1 diabetes mellitus with mild nonproliferative diabetic retinopathy without macular edema, bilateral
T1D	ICD10CM	E10.3299	Type 1 diabetes mellitus with mild nonproliferative diabetic retinopathy without macular edema, unspecified eye
T1D	ICD10CM	E10.33	Type 1 diabetes mellitus with moderate nonproliferative diabetic retinopathy
T1D	ICD10CM	E10.331	Type 1 diabetes mellitus with moderate nonproliferative diabetic retinopathy with macular edema
T1D	ICD10CM	E10.3311	Type 1 diabetes mellitus with moderate nonproliferative diabetic retinopathy with macular edema, right eye
T1D	ICD10CM	E10.3312	Type 1 diabetes mellitus with moderate nonproliferative diabetic retinopathy with macular edema, left eye
T1D	ICD10CM	E10.3313	Type 1 diabetes mellitus with moderate nonproliferative diabetic retinopathy with macular edema, bilateral
T1D	ICD10CM	E10.3319	Type 1 diabetes mellitus with moderate nonproliferative diabetic retinopathy with macular edema, unspecified eye
T1D	ICD10CM	E10.339	Type 1 diabetes mellitus with moderate nonproliferative diabetic retinopathy without macular edema
T1D	ICD10CM	E10.3391	Type 1 diabetes mellitus with moderate nonproliferative diabetic retinopathy without macular edema, right eye
T1D	ICD10CM	E10.3392	Type 1 diabetes mellitus with moderate nonproliferative diabetic retinopathy without macular edema, left eye
T1D	ICD10CM	E10.3393	Type 1 diabetes mellitus with moderate nonproliferative diabetic retinopathy without macular edema, bilateral
T1D	ICD10CM	E10.3399	Type 1 diabetes mellitus with moderate nonproliferative diabetic retinopathy without macular edema, unspecified eye
T1D	ICD10CM	E10.34	Type 1 diabetes mellitus with severe nonproliferative diabetic retinopathy
T1D	ICD10CM	E10.341	Type 1 diabetes mellitus with severe nonproliferative diabetic retinopathy with macular edema
T1D	ICD10CM	E10.3411	Type 1 diabetes mellitus with severe nonproliferative diabetic retinopathy with macular edema, right eye
T1D	ICD10CM	E10.3412	Type 1 diabetes mellitus with severe nonproliferative diabetic retinopathy with macular edema, left eye
T1D	ICD10CM	E10.3413	Type 1 diabetes mellitus with severe nonproliferative diabetic retinopathy with macular edema, bilateral

T1D	ICD10CM	E10.3419	Type 1 diabetes mellitus with severe nonproliferative diabetic retinopathy with macular edema, unspecified eye
T1D	ICD10CM	E10.349	Type 1 diabetes mellitus with severe nonproliferative diabetic retinopathy without macular edema
T1D	ICD10CM	E10.3491	Type 1 diabetes mellitus with severe nonproliferative diabetic retinopathy without macular edema, right eye
T1D	ICD10CM	E10.3492	Type 1 diabetes mellitus with severe nonproliferative diabetic retinopathy without macular edema, left eye
T1D	ICD10CM	E10.3493	Type 1 diabetes mellitus with severe nonproliferative diabetic retinopathy without macular edema, bilateral
T1D	ICD10CM	E10.3499	Type 1 diabetes mellitus with severe nonproliferative diabetic retinopathy without macular edema, unspecified eye
T1D	ICD10CM	E10.35	Type 1 diabetes mellitus with proliferative diabetic retinopathy
T1D	ICD10CM	E10.351	Type 1 diabetes mellitus with proliferative diabetic retinopathy with macular edema
T1D	ICD10CM	E10.3511	Type 1 diabetes mellitus with proliferative diabetic retinopathy with macular edema, right eye
T1D	ICD10CM	E10.3512	Type 1 diabetes mellitus with proliferative diabetic retinopathy with macular edema, left eye
T1D	ICD10CM	E10.3513	Type 1 diabetes mellitus with proliferative diabetic retinopathy with macular edema, bilateral
T1D	ICD10CM	E10.3519	Type 1 diabetes mellitus with proliferative diabetic retinopathy with macular edema, unspecified eye
T1D	ICD10CM	E10.352	Type 1 diabetes mellitus with proliferative diabetic retinopathy with traction retinal detachment involving the macula
T1D	ICD10CM	E10.3521	Type 1 diabetes mellitus with proliferative diabetic retinopathy with traction retinal detachment involving the macula, right eye
T1D	ICD10CM	E10.3522	Type 1 diabetes mellitus with proliferative diabetic retinopathy with traction retinal detachment involving the macula, left eye
T1D	ICD10CM	E10.3523	Type 1 diabetes mellitus with proliferative diabetic retinopathy with traction retinal detachment involving the macula, bilateral
T1D	ICD10CM	E10.3529	Type 1 diabetes mellitus with proliferative diabetic retinopathy with traction retinal detachment involving the macula, unspecified eye
T1D	ICD10CM	E10.353	Type 1 diabetes mellitus with proliferative diabetic retinopathy with traction retinal detachment not involving the macula
T1D	ICD10CM	E10.3531	Type 1 diabetes mellitus with proliferative diabetic retinopathy with traction retinal detachment not involving the macula, right eye

T1D	ICD10CM	E10.3532	Type 1 diabetes mellitus with proliferative diabetic retinopathy with traction retinal detachment not involving the macula, left eye
T1D	ICD10CM	E10.3533	Type 1 diabetes mellitus with proliferative diabetic retinopathy with traction retinal detachment not involving the macula, bilateral
T1D	ICD10CM	E10.3539	Type 1 diabetes mellitus with proliferative diabetic retinopathy with traction retinal detachment not involving the macula, unspecified eye
T1D	ICD10CM	E10.354	Type 1 diabetes mellitus with proliferative diabetic retinopathy with combined traction retinal detachment and rhegmatogenous retinal detachment
T1D	ICD10CM	E10.3541	Type 1 diabetes mellitus with proliferative diabetic retinopathy with combined traction retinal detachment and rhegmatogenous retinal detachment, right eye
T1D	ICD10CM	E10.3542	Type 1 diabetes mellitus with proliferative diabetic retinopathy with combined traction retinal detachment and rhegmatogenous retinal detachment, left eye
T1D	ICD10CM	E10.3543	Type 1 diabetes mellitus with proliferative diabetic retinopathy with combined traction retinal detachment and rhegmatogenous retinal detachment, bilateral
T1D	ICD10CM	E10.3549	Type 1 diabetes mellitus with proliferative diabetic retinopathy with combined traction retinal detachment and rhegmatogenous retinal detachment, unspecified eye
T1D	ICD10CM	E10.355	Type 1 diabetes mellitus with stable proliferative diabetic retinopathy
T1D	ICD10CM	E10.3551	Type 1 diabetes mellitus with stable proliferative diabetic retinopathy, right eye
T1D	ICD10CM	E10.3552	Type 1 diabetes mellitus with stable proliferative diabetic retinopathy, left eye
T1D	ICD10CM	E10.3553	Type 1 diabetes mellitus with stable proliferative diabetic retinopathy, bilateral
T1D	ICD10CM	E10.3559	Type 1 diabetes mellitus with stable proliferative diabetic retinopathy, unspecified eye
T1D	ICD10CM	E10.359	Type 1 diabetes mellitus with proliferative diabetic retinopathy without macular edema
T1D	ICD10CM	E10.3591	Type 1 diabetes mellitus with proliferative diabetic retinopathy without macular edema, right eye
T1D	ICD10CM	E10.3592	Type 1 diabetes mellitus with proliferative diabetic retinopathy without macular edema, left eye
T1D	ICD10CM	E10.3593	Type 1 diabetes mellitus with proliferative diabetic retinopathy without macular edema, bilateral
T1D	ICD10CM	E10.3599	Type 1 diabetes mellitus with proliferative diabetic retinopathy without macular edema, unspecified eye
T1D	ICD10CM	E10.36	Type 1 diabetes mellitus with diabetic cataract
T1D	ICD10CM	E10.37	Type 1 diabetes mellitus with diabetic macular edema, resolved following treatment
T1D	ICD10CM	E10.37X1	Type 1 diabetes mellitus with diabetic macular edema, resolved following treatment, right eye
T1D	ICD10CM	E10.37X2	Type 1 diabetes mellitus with diabetic macular edema, resolved following treatment, left eye

T1D	ICD10CM	E10.37X3	Type 1 diabetes mellitus with diabetic macular edema, resolved following treatment, bilateral
T1D	ICD10CM	E10.37X9	Type 1 diabetes mellitus with diabetic macular edema, resolved following treatment, unspecified eye
T1D	ICD10CM	E10.39	Type 1 diabetes mellitus with other diabetic ophthalmic complication
T1D	ICD10CM	E10.4	Type 1 diabetes mellitus with neurological complications
T1D	ICD10CM	E10.40	Type 1 diabetes mellitus with diabetic neuropathy, unspecified
T1D	ICD10CM	E10.41	Type 1 diabetes mellitus with diabetic mononeuropathy
T1D	ICD10CM	E10.42	Type 1 diabetes mellitus with diabetic polyneuropathy
T1D	ICD10CM	E10.43	Type 1 diabetes mellitus with diabetic autonomic (poly)neuropathy
T1D	ICD10CM	E10.44	Type 1 diabetes mellitus with diabetic amyotrophy
T1D	ICD10CM	E10.49	Type 1 diabetes mellitus with other diabetic neurological complication
T1D	ICD10CM	E10.5	Type 1 diabetes mellitus with circulatory complications
T1D	ICD10CM	E10.51	Type 1 diabetes mellitus with diabetic peripheral angiopathy without gangrene
T1D	ICD10CM	E10.52	Type 1 diabetes mellitus with diabetic peripheral angiopathy with gangrene
T1D	ICD10CM	E10.59	Type 1 diabetes mellitus with other circulatory complications
T1D	ICD10CM	E10.6	Type 1 diabetes mellitus with other specified complications
T1D	ICD10CM	E10.61	Type 1 diabetes mellitus with diabetic arthropathy
T1D	ICD10CM	E10.610	Type 1 diabetes mellitus with diabetic neuropathic arthropathy
T1D	ICD10CM	E10.618	Type 1 diabetes mellitus with other diabetic arthropathy
T1D	ICD10CM	E10.62	Type 1 diabetes mellitus with skin complications
T1D	ICD10CM	E10.620	Type 1 diabetes mellitus with diabetic dermatitis
T1D	ICD10CM	E10.621	Type 1 diabetes mellitus with foot ulcer
T1D	ICD10CM	E10.622	Type 1 diabetes mellitus with other skin ulcer
T1D	ICD10CM	E10.628	Type 1 diabetes mellitus with other skin complications
T1D	ICD10CM	E10.63	Type 1 diabetes mellitus with oral complications
T1D	ICD10CM	E10.630	Type 1 diabetes mellitus with periodontal disease
T1D	ICD10CM	E10.638	Type 1 diabetes mellitus with other oral complications
T1D	ICD10CM	E10.64	Type 1 diabetes mellitus with hypoglycemia
T1D	ICD10CM	E10.640	Type 1 diabetes mellitus with hypoglycemia without coma

T1D	ICD10CM	E10.641	Type 1 diabetes mellitus with hypoglycemia with coma
T1D	ICD10CM	E10.649	Type 1 diabetes mellitus with hypoglycemia without coma
T1D	ICD10CM	E10.65	Type 1 diabetes mellitus with hyperglycemia
T1D	ICD10CM	E10.69	Type 1 diabetes mellitus with other specified complication
T1D	ICD10CM	E10.8	Type 1 diabetes mellitus with unspecified complications
T1D	ICD10CM	E10.9	Type 1 diabetes mellitus without complications
T1D	ICD9CM	250.01	Diabetes mellitus without mention of complication, type I [juvenile type], not stated as uncontrolled
T1D	ICD9CM	250.03	Diabetes mellitus without mention of complication, type I [juvenile type], uncontrolled
T1D	ICD9CM	250.11	Diabetes with ketoacidosis, type I [juvenile type], not stated as uncontrolled
T1D	ICD9CM	250.13	Diabetes with ketoacidosis, type I [juvenile type], uncontrolled
T1D	ICD9CM	250.21	Diabetes with hyperosmolarity, type I [juvenile type], not stated as uncontrolled
T1D	ICD9CM	250.23	Diabetes with hyperosmolarity, type I [juvenile type], uncontrolled
T1D	ICD9CM	250.31	Diabetes with other coma, type I [juvenile type], not stated as uncontrolled
T1D	ICD9CM	250.33	Diabetes with other coma, type I [juvenile type], uncontrolled
T1D	ICD9CM	250.41	Diabetes with renal manifestations, type I [juvenile type], not stated as uncontrolled
T1D	ICD9CM	250.43	Diabetes with renal manifestations, type I [juvenile type], uncontrolled
T1D	ICD9CM	250.51	Diabetes with ophthalmic manifestations, type I [juvenile type], not stated as uncontrolled
T1D	ICD9CM	250.53	Diabetes with ophthalmic manifestations, type I [juvenile type], uncontrolled
T1D	ICD9CM	250.61	Diabetes with neurological manifestations, type I [juvenile type], not stated as uncontrolled
T1D	ICD9CM	250.63	Diabetes with neurological manifestations, type I [juvenile type], uncontrolled
T1D	ICD9CM	250.71	Diabetes with peripheral circulatory disorders, type I [juvenile type], not stated as uncontrolled
T1D	ICD9CM	250.73	Diabetes with peripheral circulatory disorders, type I [juvenile type], uncontrolled
T1D	ICD9CM	250.81	Diabetes with other specified manifestations, type I [juvenile type], not stated as uncontrolled
T1D	ICD9CM	250.83	Diabetes with other specified manifestations, type I [juvenile type], uncontrolled
T1D	ICD9CM	250.91	Diabetes with unspecified complication, type I [juvenile type], not stated as uncontrolled
T1D	ICD9CM	250.93	Diabetes with unspecified complication, type I [juvenile type], uncontrolled
T2D	ICD10CM	E11	Type 2 diabetes mellitus
T2D	ICD10CM	E11.0	Type 2 diabetes mellitus with hyperosmolarity

T2D	ICD10CM	E11.00	Type 2 diabetes mellitus with hyperosmolarity without nonketotic hyperglycaemic-hyperosmolar coma (NKHHC)
T2D	ICD10CM	E11.01	Type 2 diabetes mellitus with hyperosmolarity with coma
T2D	ICD10CM	E11.1	Type 2 diabetes mellitus with ketoacidosis
T2D	ICD10CM	E11.10	Type 2 diabetes mellitus with ketoacidosis without coma
T2D	ICD10CM	E11.11	Type 2 diabetes mellitus with ketoacidosis with coma
T2D	ICD10CM	E11.2	Type 2 diabetes mellitus with kidney complications
T2D	ICD10CM	E11.21	Type 2 diabetes mellitus with diabetic nephropathy
T2D	ICD10CM	E11.22	Type 2 diabetes mellitus with diabetic chronic kidney disease
T2D	ICD10CM	E11.29	Type 2 diabetes mellitus with other diabetic kidney complication
T2D	ICD10CM	E11.3	Type 2 diabetes mellitus with ophthalmic complications
T2D	ICD10CM	E11.31	Type 2 diabetes mellitus with unspecified diabetic retinopathy
T2D	ICD10CM	E11.311	Type 2 diabetes mellitus with unspecified diabetic retinopathy with macular edema
T2D	ICD10CM	E11.319	Type 2 diabetes mellitus with unspecified diabetic retinopathy without macular edema
T2D	ICD10CM	E11.32	Type 2 diabetes mellitus with mild nonproliferative diabetic retinopathy
T2D	ICD10CM	E11.321	Type 2 diabetes mellitus with mild nonproliferative diabetic retinopathy with macular edema
T2D	ICD10CM	E11.3211	Type 2 diabetes mellitus with mild nonproliferative diabetic retinopathy with macular edema, right eye
T2D	ICD10CM	E11.3212	Type 2 diabetes mellitus with mild nonproliferative diabetic retinopathy with macular edema, left eye
T2D	ICD10CM	E11.3213	Type 2 diabetes mellitus with mild nonproliferative diabetic retinopathy with macular edema, bilateral
T2D	ICD10CM	E11.3219	Type 2 diabetes mellitus with mild nonproliferative diabetic retinopathy with macular edema, unspecified eye
T2D	ICD10CM	E11.329	Type 2 diabetes mellitus with mild nonproliferative diabetic retinopathy without macular edema
T2D	ICD10CM	E11.3291	Type 2 diabetes mellitus with mild nonproliferative diabetic retinopathy without macular edema, right eye
T2D	ICD10CM	E11.3292	Type 2 diabetes mellitus with mild nonproliferative diabetic retinopathy without macular edema, left eye

T2D	ICD10CM	E11.3293	Type 2 diabetes mellitus with mild nonproliferative diabetic retinopathy without macular edema, bilateral
T2D	ICD10CM	E11.3299	Type 2 diabetes mellitus with mild nonproliferative diabetic retinopathy without macular edema, unspecified eye
T2D	ICD10CM	E11.33	Type 2 diabetes mellitus with moderate nonproliferative diabetic retinopathy
T2D	ICD10CM	E11.331	Type 2 diabetes mellitus with moderate nonproliferative diabetic retinopathy with macular edema
T2D	ICD10CM	E11.3311	Type 2 diabetes mellitus with moderate nonproliferative diabetic retinopathy with macular edema, right eye
T2D	ICD10CM	E11.3312	Type 2 diabetes mellitus with moderate nonproliferative diabetic retinopathy with macular edema, left eye
T2D	ICD10CM	E11.3313	Type 2 diabetes mellitus with moderate nonproliferative diabetic retinopathy with macular edema, bilateral
T2D	ICD10CM	E11.3319	Type 2 diabetes mellitus with moderate nonproliferative diabetic retinopathy with macular edema, unspecified eye
T2D	ICD10CM	E11.339	Type 2 diabetes mellitus with moderate nonproliferative diabetic retinopathy without macular edema
T2D	ICD10CM	E11.3391	Type 2 diabetes mellitus with moderate nonproliferative diabetic retinopathy without macular edema, right eye
T2D	ICD10CM	E11.3392	Type 2 diabetes mellitus with moderate nonproliferative diabetic retinopathy without macular edema, left eye
T2D	ICD10CM	E11.3393	Type 2 diabetes mellitus with moderate nonproliferative diabetic retinopathy without macular edema, bilateral
T2D	ICD10CM	E11.3399	Type 2 diabetes mellitus with moderate nonproliferative diabetic retinopathy without macular edema, unspecified eye
T2D	ICD10CM	E11.34	Type 2 diabetes mellitus with severe nonproliferative diabetic retinopathy
T2D	ICD10CM	E11.341	Type 2 diabetes mellitus with severe nonproliferative diabetic retinopathy with macular edema
T2D	ICD10CM	E11.3411	Type 2 diabetes mellitus with severe nonproliferative diabetic retinopathy with macular edema, right eye
T2D	ICD10CM	E11.3412	Type 2 diabetes mellitus with severe nonproliferative diabetic retinopathy with macular edema, left eye
T2D	ICD10CM	E11.3413	Type 2 diabetes mellitus with severe nonproliferative diabetic retinopathy with macular edema, bilateral

T2D	ICD10CM	E11.3419	Type 2 diabetes mellitus with severe nonproliferative diabetic retinopathy with macular edema, unspecified eye
T2D	ICD10CM	E11.349	Type 2 diabetes mellitus with severe nonproliferative diabetic retinopathy without macular edema
T2D	ICD10CM	E11.3491	Type 2 diabetes mellitus with severe nonproliferative diabetic retinopathy without macular edema, right eye
T2D	ICD10CM	E11.3492	Type 2 diabetes mellitus with severe nonproliferative diabetic retinopathy without macular edema, left eye
T2D	ICD10CM	E11.3493	Type 2 diabetes mellitus with severe nonproliferative diabetic retinopathy without macular edema, bilateral
T2D	ICD10CM	E11.3499	Type 2 diabetes mellitus with severe nonproliferative diabetic retinopathy without macular edema, unspecified eye
T2D	ICD10CM	E11.35	Type 2 diabetes mellitus with proliferative diabetic retinopathy
T2D	ICD10CM	E11.351	Type 2 diabetes mellitus with proliferative diabetic retinopathy with macular edema
T2D	ICD10CM	E11.3511	Type 2 diabetes mellitus with proliferative diabetic retinopathy with macular edema, right eye
T2D	ICD10CM	E11.3512	Type 2 diabetes mellitus with proliferative diabetic retinopathy with macular edema, left eye
T2D	ICD10CM	E11.3513	Type 2 diabetes mellitus with proliferative diabetic retinopathy with macular edema, bilateral
T2D	ICD10CM	E11.3519	Type 2 diabetes mellitus with proliferative diabetic retinopathy with macular edema, unspecified eye
T2D	ICD10CM	E11.352	Type 2 diabetes mellitus with proliferative diabetic retinopathy with traction retinal detachment involving the macula
T2D	ICD10CM	E11.3521	Type 2 diabetes mellitus with proliferative diabetic retinopathy with traction retinal detachment involving the macula, right eye
T2D	ICD10CM	E11.3522	Type 2 diabetes mellitus with proliferative diabetic retinopathy with traction retinal detachment involving the macula, left eye
T2D	ICD10CM	E11.3523	Type 2 diabetes mellitus with proliferative diabetic retinopathy with traction retinal detachment involving the macula, bilateral
T2D	ICD10CM	E11.3529	Type 2 diabetes mellitus with proliferative diabetic retinopathy with traction retinal detachment involving the macula, unspecified eye
T2D	ICD10CM	E11.353	Type 2 diabetes mellitus with proliferative diabetic retinopathy with traction retinal detachment not involving the macula
T2D	ICD10CM	E11.3531	Type 2 diabetes mellitus with proliferative diabetic retinopathy with traction retinal detachment not involving the macula, right eye

T2D	ICD10CM	E11.3532	Type 2 diabetes mellitus with proliferative diabetic retinopathy with traction retinal detachment not involving the macula, left eye
T2D	ICD10CM	E11.3533	Type 2 diabetes mellitus with proliferative diabetic retinopathy with traction retinal detachment not involving the macula, bilateral
T2D	ICD10CM	E11.3539	Type 2 diabetes mellitus with proliferative diabetic retinopathy with traction retinal detachment not involving the macula, unspecified eye
T2D	ICD10CM	E11.354	Type 2 diabetes mellitus with proliferative diabetic retinopathy with combined traction retinal detachment and rhegmatogenous retinal detachment
T2D	ICD10CM	E11.3541	Type 2 diabetes mellitus with proliferative diabetic retinopathy with combined traction retinal detachment and rhegmatogenous retinal detachment, right eye
T2D	ICD10CM	E11.3542	Type 2 diabetes mellitus with proliferative diabetic retinopathy with combined traction retinal detachment and rhegmatogenous retinal detachment, left eye
T2D	ICD10CM	E11.3543	Type 2 diabetes mellitus with proliferative diabetic retinopathy with combined traction retinal detachment and rhegmatogenous retinal detachment, bilateral
T2D	ICD10CM	E11.3549	Type 2 diabetes mellitus with proliferative diabetic retinopathy with combined traction retinal detachment and rhegmatogenous retinal detachment, unspecified eye
T2D	ICD10CM	E11.355	Type 2 diabetes mellitus with stable proliferative diabetic retinopathy
T2D	ICD10CM	E11.3551	Type 2 diabetes mellitus with stable proliferative diabetic retinopathy, right eye
T2D	ICD10CM	E11.3552	Type 2 diabetes mellitus with stable proliferative diabetic retinopathy, left eye
T2D	ICD10CM	E11.3553	Type 2 diabetes mellitus with stable proliferative diabetic retinopathy, bilateral
T2D	ICD10CM	E11.3559	Type 2 diabetes mellitus with stable proliferative diabetic retinopathy, unspecified eye
T2D	ICD10CM	E11.359	Type 2 diabetes mellitus with proliferative diabetic retinopathy without macular edema
T2D	ICD10CM	E11.3591	Type 2 diabetes mellitus with proliferative diabetic retinopathy without macular edema, right eye
T2D	ICD10CM	E11.3592	Type 2 diabetes mellitus with proliferative diabetic retinopathy without macular edema, left eye
T2D	ICD10CM	E11.3593	Type 2 diabetes mellitus with proliferative diabetic retinopathy without macular edema, bilateral
T2D	ICD10CM	E11.3599	Type 2 diabetes mellitus with proliferative diabetic retinopathy without macular edema, unspecified eye
T2D	ICD10CM	E11.36	Type 2 diabetes mellitus with diabetic cataract
T2D	ICD10CM	E11.37	Type 2 diabetes mellitus with diabetic macular edema, resolved following treatment
T2D	ICD10CM	E11.37X1	Type 2 diabetes mellitus with diabetic macular edema, resolved following treatment, right eye
T2D	ICD10CM	E11.37X2	Type 2 diabetes mellitus with diabetic macular edema, resolved following treatment, left eye

T2D	ICD10CM	E11.37X3	Type 2 diabetes mellitus with diabetic macular edema, resolved following treatment, bilateral
T2D	ICD10CM	E11.37X9	Type 2 diabetes mellitus with diabetic macular edema, resolved following treatment, unspecified eye
T2D	ICD10CM	E11.39	Type 2 diabetes mellitus with other diabetic ophthalmic complication
T2D	ICD10CM	E11.4	Type 2 diabetes mellitus with neurological complications
T2D	ICD10CM	E11.40	Type 2 diabetes mellitus with diabetic neuropathy, unspecified
T2D	ICD10CM	E11.41	Type 2 diabetes mellitus with diabetic mononeuropathy
T2D	ICD10CM	E11.42	Type 2 diabetes mellitus with diabetic polyneuropathy
T2D	ICD10CM	E11.43	Type 2 diabetes mellitus with diabetic autonomic (poly)neuropathy
T2D	ICD10CM	E11.44	Type 2 diabetes mellitus with diabetic amyotrophy
T2D	ICD10CM	E11.49	Type 2 diabetes mellitus with other diabetic neurological complication
T2D	ICD10CM	E11.5	Type 2 diabetes mellitus with circulatory complications
T2D	ICD10CM	E11.51	Type 2 diabetes mellitus with diabetic peripheral angiopathy without gangrene
T2D	ICD10CM	E11.52	Type 2 diabetes mellitus with diabetic peripheral angiopathy with gangrene
T2D	ICD10CM	E11.59	Type 2 diabetes mellitus with other circulatory complications
T2D	ICD10CM	E11.6	Type 2 diabetes mellitus with other specified complications
T2D	ICD10CM	E11.61	Type 2 diabetes mellitus with diabetic arthropathy
T2D	ICD10CM	E11.610	Type 2 diabetes mellitus with diabetic neuropathic arthropathy
T2D	ICD10CM	E11.618	Type 2 diabetes mellitus with other diabetic arthropathy
T2D	ICD10CM	E11.62	Type 2 diabetes mellitus with skin complications
T2D	ICD10CM	E11.620	Type 2 diabetes mellitus with diabetic dermatitis
T2D	ICD10CM	E11.621	Type 2 diabetes mellitus with foot ulcer
T2D	ICD10CM	E11.622	Type 2 diabetes mellitus with other skin ulcer
T2D	ICD10CM	E11.628	Type 2 diabetes mellitus with other skin complications
T2D	ICD10CM	E11.63	Type 2 diabetes mellitus with oral complications
T2D	ICD10CM	E11.630	Type 2 diabetes mellitus with periodontal disease
T2D	ICD10CM	E11.638	Type 2 diabetes mellitus with other oral complications
T2D	ICD10CM	E11.64	Type 2 diabetes mellitus with hypoglycemia
T2D	ICD10CM	E11.640	Type 2 diabetes mellitus with hypoglycemia without coma

T2D	ICD10CM	E11.641	Type 2 diabetes mellitus with hypoglycemia with coma
T2D	ICD10CM	E11.649	Type 2 diabetes mellitus with hypoglycemia without coma
T2D	ICD10CM	E11.65	Type 2 diabetes mellitus with hyperglycemia
T2D	ICD10CM	E11.69	Type 2 diabetes mellitus with other specified complication
T2D	ICD10CM	E11.8	Type 2 diabetes mellitus with unspecified complications
T2D	ICD10CM	E11.9	Type 2 diabetes mellitus without complications
T2D	ICD9CM	250.00	Diabetes mellitus without mention of complication, type II or unspecified type, not stated as uncontrolled
T2D	ICD9CM	250.02	Diabetes mellitus without mention of complication, type II or unspecified type, uncontrolled
T2D	ICD9CM	250.10	Diabetes with ketoacidosis, type II or unspecified type, not stated as uncontrolled
T2D	ICD9CM	250.12	Diabetes with ketoacidosis, type II or unspecified type, uncontrolled
T2D	ICD9CM	250.20	Diabetes with hyperosmolarity, type II or unspecified type, not stated as uncontrolled
T2D	ICD9CM	250.22	Diabetes with hyperosmolarity, type II or unspecified type, uncontrolled
T2D	ICD9CM	250.30	Diabetes with other coma, type II or unspecified type, not stated as uncontrolled
T2D	ICD9CM	250.32	Diabetes with other coma, type II or unspecified type, uncontrolled
T2D	ICD9CM	250.40	Diabetes with renal manifestations, type II or unspecified type, not stated as uncontrolled
T2D	ICD9CM	250.42	Diabetes with renal manifestations, type II or unspecified type, uncontrolled
T2D	ICD9CM	250.50	Diabetes with ophthalmic manifestations, type II or unspecified type, not stated as uncontrolled
T2D	ICD9CM	250.52	Diabetes with ophthalmic manifestations, type II or unspecified type, uncontrolled
T2D	ICD9CM	250.60	Diabetes with neurological manifestations, type II or unspecified type, not stated as uncontrolled
T2D	ICD9CM	250.62	Diabetes with neurological manifestations, type II or unspecified type, uncontrolled
T2D	ICD9CM	250.70	Diabetes with peripheral circulatory disorders, type II or unspecified type, not stated as uncontrolled
T2D	ICD9CM	250.72	Diabetes with peripheral circulatory disorders, type II or unspecified type, uncontrolled
T2D	ICD9CM	250.80	Diabetes with other specified manifestations, type II or unspecified type, not stated as uncontrolled
T2D	ICD9CM	250.82	Diabetes with other specified manifestations, type II or unspecified type, uncontrolled
T2D	ICD9CM	250.90	Diabetes with unspecified complication, type II or unspecified type, not stated as uncontrolled
T2D	ICD9CM	250.92	Diabetes with unspecified complication, type II or unspecified type, uncontrolled
Angina Pectoris	ICD10CM	I20.1	Angina pectoris with documented spasm
Angina Pectoris	ICD10CM	I20.8	Other forms of angina pectoris

Angina Pectoris	ICD10CM	I20.9	Angina pectoris, unspecified
Angina Pectoris	ICD9CM	440.2	Atherosclerosis of native arteries of the extremities
Angina Pectoris	ICD9CM	440.4	Chronic total occlusion of artery of the extremities
Angina Pectoris	ICD9CM	443.9	Peripheral vascular disease, unspecified
ASCVD	ICD10CM	G45	Transient cerebral ischemic attacks and related syndromes
ASCVD	ICD10CM	I20	Angina pectoris
ASCVD	ICD10CM	I21	Acute myocardial infarction
ASCVD	ICD10CM	I22	Subsequent ST elevation (STEMI) and non-ST elevation (NSTEMI) myocardial infarction
ASCVD	ICD10CM	I23	Certain current complications following ST elevation (STEMI) and non-ST elevation (NSTEMI) myocardial infarction (within the 28 day period)
ASCVD	ICD10CM	I24	Other acute ischemic heart diseases
ASCVD	ICD10CM	I25	Chronic ischemic heart disease
ASCVD	ICD10CM	I60	Nontraumatic subarachnoid hemorrhage
ASCVD	ICD10CM	I61	Nontraumatic intracerebral hemorrhage
ASCVD	ICD10CM	I62	Other and unspecified nontraumatic intracranial hemorrhage
ASCVD	ICD10CM	I63	Cerebral infarction
ASCVD	ICD10CM	I65	Occlusion and stenosis of precerebral arteries, not resulting in cerebral infarction
ASCVD	ICD10CM	I66	Occlusion and stenosis of cerebral arteries, not resulting in cerebral infarction
ASCVD	ICD10CM	I67	Other cerebrovascular diseases
ASCVD	ICD10CM	I68	Cerebrovascular disorders in diseases classified elsewhere
ASCVD	ICD10CM	I69	Sequelae of cerebrovascular disease
ASCVD	ICD10CM	I70	Atherosclerosis
ASCVD	ICD10CM	I73.9	Peripheral vascular disease, unspecified
ASCVD	ICD10CM	Z95	Presence of cardiac and vascular implants and grafts
ASCVD	ICD9CM	410	Acute myocardial infarction
ASCVD	ICD9CM	411	Other acute and subacute forms of ischemic heart disease
ASCVD	ICD9CM	412	Old myocardial infarction
ASCVD	ICD9CM	413	Angina pectoris
ASCVD	ICD9CM	414	Other forms of chronic ischemic heart disease

ASCVD	ICD9CM	430	Subarachnoid hemorrhage
ASCVD	ICD9CM	431	Intracerebral hemorrhage
ASCVD	ICD9CM	432	Other and unspecified intracranial hemorrhage
ASCVD	ICD9CM	433	Occlusion and stenosis of precerebral arteries
ASCVD	ICD9CM	434	Occlusion of cerebral arteries
ASCVD	ICD9CM	435	Transient cerebral ischemia
ASCVD	ICD9CM	436	Acute, but ill-defined, cerebrovascular disease
ASCVD	ICD9CM	437	Other and ill-defined cerebrovascular disease
ASCVD	ICD9CM	438	Late effects of cerebrovascular disease
Diabetic foot	ICD10CM	E10.5	Type 1 diabetes mellitus with circulatory complications
Diabetic foot	ICD10CM	E11.5	Type 2 diabetes mellitus with circulatory complications
Diabetic foot	ICD9CM	440.24	Atherosclerosis of native arteries of the extremities with gangrene
Established CVD	ICD10CM	H34.1	Central retinal artery occlusion
Established CVD	ICD10CM	H34.2	Other retinal artery occlusions
Established CVD	ICD10CM	I21	Acute myocardial infarction
Established CVD	ICD10CM	I22	Subsequent ST elevation (STEMI) and non-ST elevation (NSTEMI) myocardial infarction
Established CVD	ICD10CM	I25.1	Atherosclerotic heart disease of native coronary artery
Established CVD	ICD10CM	I25.2	Old myocardial infarction
Established CVD	ICD10CM	I61	Nontraumatic intracerebral hemorrhage
Established CVD	ICD10CM	I63	Cerebral infarction
Established CVD	ICD9CM	410	Acute myocardial infarction
Established CVD	ICD9CM	412	Old myocardial infarction
Established CVD	ICD9CM	429.9	Heart disease, unspecified
Established CVD	ICD9CM	431	Intracerebral hemorrhage
Established CVD	ICD9CM	433	Occlusion and stenosis of precerebral arteries
Established CVD	ICD9CM	434	Occlusion of cerebral arteries
Established CVD	ICD9CM	435	Transient cerebral ischemia
Heart failure	ICD10CM	I11	Hypertensive heart disease
Heart failure	ICD10CM	I13	Hypertensive heart and chronic kidney disease

Heart failure	ICD10CM	I25.5	Ischemic cardiomyopathy
Heart failure	ICD10CM	I25.6	Silent myocardial ischemia
Heart failure	ICD10CM	I42.0	Dilated cardiomyopathy
Heart failure	ICD10CM	I42.5	Other restrictive cardiomyopathy
Heart failure	ICD10CM	I42.6	Alcoholic cardiomyopathy
Heart failure	ICD10CM	I42.7	Cardiomyopathy due to drug and external agent
Heart failure	ICD10CM	I42.8	Other cardiomyopathies
Heart failure	ICD10CM	I42.9	Cardiomyopathy, unspecified
Heart failure	ICD10CM	I50	Heart failure
Heart failure	ICD9CM	428	Heart failure
History of CKD	ICD10CM	E10.2	Type 1 diabetes mellitus with kidney complications
History of CKD	ICD10CM	E11.2	Type 2 diabetes mellitus with kidney complications
History of CKD	ICD10CM	I12	Hypertensive chronic kidney disease
History of CKD	ICD10CM	I12.0	Hypertensive chronic kidney disease with stage 5 chronic kidney disease or end stage renal disease
History of CKD	ICD10CM	I12.9	Hypertensive chronic kidney disease with stage 1 through stage 4 chronic kidney disease, or unspecified chronic kidney disease
History of CKD	ICD10CM	I13	Hypertensive heart and chronic kidney disease
History of CKD	ICD10CM	N18	Chronic kidney disease (CKD)
History of CKD	ICD10CM	N19	Unspecified kidney failure
History of CKD	ICD10CM	N25	Disorders resulting from impaired renal tubular function
History of CKD	ICD10CM	Z49	Encounter for care involving renal dialysis
History of CKD	ICD9CM	250.42	Diabetes with renal manifestations, type II or unspecified type, uncontrolled
History of CKD	ICD9CM	583	Nephritis and nephropathy, not specified as acute or chronic
History of CKD	ICD9CM	584	Acute kidney failure
History of CKD	ICD9CM	585	Chronic kidney disease (CKD)
History of CKD	ICD9CM	586	Renal failure, unspecified
Hypertension	ICD10CM	I10	Essential (primary) hypertension
Hypertension	ICD10CM	I11.9	Hypertensive heart disease without heart failure
Hypertension	ICD9CM	401.0	Malignant essential hypertension

Hypertension	ICD9CM	401.1	Benign essential hypertension
Hypertension	ICD9CM	401.9	Unspecified essential hypertension
Myocardial infarction	ICD10CM	I21	Acute myocardial infarction
Myocardial infarction	ICD10CM	I22	Subsequent ST elevation (STEMI) and non-ST elevation (NSTEMI) myocardial infarction
Myocardial infarction	ICD10CM	I25.2	Old myocardial infarction
Myocardial infarction	ICD9CM	410	Acute myocardial infarction
Neuropathy	ICD10CM	E10.4	Type 1 diabetes mellitus with neurological complications
Neuropathy	ICD10CM	E11.4	Type 2 diabetes mellitus with neurological complications
Neuropathy	ICD9CM	250.60	Diabetes with neurological manifestations, type II or unspecified type, not stated as uncontrolled
Neuropathy	ICD9CM	338	Pain, not elsewhere classified
Neuropathy	ICD9CM	354	Mononeuritis of upper limb and mononeuritis multiplex
Neuropathy	ICD9CM	355	Mononeuritis of lower limb
Neuropathy	ICD9CM	356.9	Unspecified hereditary and idiopathic peripheral neuropathy
Neuropathy	ICD9CM	357.2	Polyneuropathy in diabetes
Obesity	ICD10CM	E66	Overweight and obesity
Obesity	ICD10CM	Z68.3	Body mass index (BMI) 30-39, adult
Obesity	ICD10CM	Z68.4	Body mass index (BMI) 40 or greater, adult
Obesity	ICD10CM	Z68.4	Body mass index (BMI) 40 or greater, adult
Obesity	ICD9CM	278	Overweight, obesity and other hyperalimentation
Obesity	ICD9CM	V85.30	Body Mass Index 30.0–30.9, adult
Obesity	ICD9CM	V85.41	Body Mass Index 40.0–44.9, adult
Obesity	ICD9CM	V85.42	Body Mass Index 45.0–49.9, adult
Obesity	ICD9CM	V85.43	Body Mass Index 50.0–59.9, adult
Obesity	ICD9CM	V85.44	Body Mass Index 60.0–69.9, adult
Obesity	ICD9CM	V85.45	Body Mass Index 70 and over, adult
Obesity	ICD9CM	V85.54	Body Mass Index, pediatric, greater than or equal to 95th percentile for age

Peripheral vascular disease	ICD10CM	E11.5	Type 2 diabetes mellitus with circulatory complications
Peripheral vascular disease	ICD10CM	I70.2	Atherosclerosis of native arteries of the extremities
Peripheral vascular disease	ICD10CM	I73.1	Thromboangiitis obliterans [Buerger's disease]
Peripheral vascular disease	ICD10CM	I73.8	Other specified peripheral vascular diseases
Peripheral vascular disease	ICD10CM	I73.9	Peripheral vascular disease, unspecified
Peripheral vascular disease	ICD10CM	I77.1	Stricture of artery
Peripheral vascular disease	ICD10CM	I79.0	Aneurysm of aorta in diseases classified elsewhere
Peripheral vascular disease	ICD10CM	I79.1	Aortitis in diseases classified elsewhere
Peripheral vascular disease	ICD10CM	I79.8	Other disorders of arteries, arterioles and capillaries in diseases classified elsewhere
Peripheral vascular disease	ICD10CM	K55.1	Chronic vascular disorders of intestine
Peripheral vascular disease	ICD10CM	K55.8	Other vascular disorders of intestine
Peripheral vascular disease	ICD10CM	K55.9	Vascular disorder of intestine, unspecified
Peripheral vascular disease	ICD10CM	Z95.8	Presence of other cardiac and vascular implants and grafts
Peripheral vascular disease	ICD10CM	Z95.9	Presence of cardiac and vascular implant and graft, unspecified
Peripheral vascular disease	ICD9CM	440.2	Atherosclerosis of native arteries of the extremities
Peripheral vascular disease	ICD9CM	440.4	Chronic total occlusion of artery of the extremities

Peripheral vascular disease	ICD9CM	443.9	Peripheral vascular disease, unspecified
Retinopathy	ICD10CM	E10.3	Type 1 diabetes mellitus with ophthalmic complications
Retinopathy	ICD10CM	E11.3	Type 2 diabetes mellitus with ophthalmic complications
Retinopathy	ICD10CM	H31	Other disorders of choroid
Retinopathy	ICD10CM	H32	Chorioretinal disorders in diseases classified elsewhere
Retinopathy	ICD10CM	H33	Retinal detachments and breaks
Retinopathy	ICD10CM	H34	Retinal vascular occlusions
Retinopathy	ICD10CM	H35	Other retinal disorders
Retinopathy	ICD10CM	H36	Retinal disorders in diseases classified elsewhere
Retinopathy	ICD9CM	250.50	Diabetes with ophthalmic manifestations, type II or unspecified type, not stated as uncontrolled
Retinopathy	ICD9CM	362.0	Diabetic retinopathy
Retinopathy	ICD9CM	379.22	Crystalline deposits in vitreous
Retinopathy	ICD9CM	379.23	Vitreous hemorrhage
Stroke	ICD10CM	G45	Transient cerebral ischemic attacks and related syndromes
Stroke	ICD10CM	G46	Vascular syndromes of brain in cerebrovascular diseases
Stroke	ICD10CM	H34.1	Central retinal artery occlusion
Stroke	ICD10CM	H34.2	Other retinal artery occlusions
Stroke	ICD10CM	I61	Nontraumatic intracerebral hemorrhage
Stroke	ICD10CM	I63	Cerebral infarction
Stroke	ICD9CM	431	Intracerebral hemorrhage
Stroke	ICD9CM	433	Occlusion and stenosis of precerebral arteries
Stroke	ICD9CM	434	Occlusion of cerebral arteries
Stroke	ICD9CM	435	Transient cerebral ischemia

CKD, chronic kidney disease; ICD9CM, International Classification of Diseases, Ninth Revision, Clinical Modification; ICD10CM, International Classification of Diseases, Tenth Revision, Clinical Modification; T1D, type 1 diabetes; T2D, type 2 diabetes.

Supplementary Table S2. National Drug Codes used to identify GLP-1 RA users from the Optum® database

Codelist name	NDC	Generic name	Description
GLP-1 RA	00173086601	ALBIGLUTIDE	TANZEUM 30 MG/0.5 ML SUBCUTANEOUS PEN INJECTOR
GLP-1 RA	00173086602	ALBIGLUTIDE	TANZEUM 30 MG/0.5 ML SUBCUTANEOUS PEN INJECTOR
GLP-1 RA	00173086635	ALBIGLUTIDE	TANZEUM 30 MG/0.5 ML SUBCUTANEOUS PEN INJECTOR
GLP-1 RA	00173086701	ALBIGLUTIDE	TANZEUM 50 MG/0.5 ML SUBCUTANEOUS PEN INJECTOR
GLP-1 RA	00173086702	ALBIGLUTIDE	TANZEUM 50 MG/0.5 ML SUBCUTANEOUS PEN INJECTOR
GLP-1 RA	00173086735	ALBIGLUTIDE	TANZEUM 50 MG/0.5 ML SUBCUTANEOUS PEN INJECTOR
GLP-1 RA	00002143301	DULAGLUTIDE	TRULICITY 0.75 MG/0.5 ML SUBCUTANEOUS PEN INJECTOR
GLP-1 RA	00002143361	DULAGLUTIDE	TRULICITY 0.75 MG/0.5 ML SUBCUTANEOUS PEN INJECTOR
GLP-1 RA	00002143380	DULAGLUTIDE	TRULICITY 0.75 MG/0.5 ML SUBCUTANEOUS PEN INJECTOR
GLP-1 RA	00002143401	DULAGLUTIDE	TRULICITY 1.5 MG/0.5 ML SUBCUTANEOUS PEN INJECTOR
GLP-1 RA	00002143480	DULAGLUTIDE	TRULICITY 1.5 MG/0.5 ML SUBCUTANEOUS PEN INJECTOR
GLP-1 RA	54868538401	EXENATIDE	BYETTA 10 MCG/0.04 ML PER DOSE SUB-Q PEN INJECTOR
GLP-1 RA	66780021008	EXENATIDE	BYETTA 10 MCG/0.04 ML PER DOSE SUB-Q PEN INJECTOR
GLP-1 RA	00310652401	EXENATIDE	BYETTA 10 MCG/DOSE (250 MCG/ML)2.4 ML SUBCUTANEOUS PEN INJECTOR
GLP-1 RA	66780021201	EXENATIDE	BYETTA 10 MCG/DOSE (250 MCG/ML)2.4 ML SUBCUTANEOUS PEN INJECTOR
GLP-1 RA	54868538400	EXENATIDE	BYETTA 5 MCG/0.02 ML PER DOSE SUB-Q PEN INJECTOR
GLP-1 RA	00310651201	EXENATIDE	BYETTA 5 MCG/DOSE (250 MCG/ML)1.2 ML SUBCUTANEOUS PEN INJECTOR
GLP-1 RA	66780021007	EXENATIDE	BYETTA 5 MCG/DOSE (250 MCG/ML)1.2 ML SUBCUTANEOUS PEN INJECTOR
GLP-1 RA	00310652004	EXENATIDE MICROSPHERES	BYDUREON 2 MG SUBCUTANEOUS EXTENDED RELEASE SUSPENSION
GLP-1 RA	66780021904	EXENATIDE MICROSPHERES	BYDUREON 2 MG SUBCUTANEOUS EXTENDED RELEASE SUSPENSION
GLP-1 RA	66780022601	EXENATIDE MICROSPHERES	BYDUREON 2 MG SUBCUTANEOUS EXTENDED RELEASE SUSPENSION
GLP-1 RA	00310653001	EXENATIDE MICROSPHERES	BYDUREON 2 MG/0.65 ML SUBCUTANEOUS PEN INJECTOR

GLP-1 RA	00310653004	EXENATIDE MICROSPHERES	BYDUREON 2 MG/0.65 ML SUBCUTANEOUS PEN INJECTOR
GLP-1 RA	00310654001	EXENATIDE MICROSPHERES	BYDUREON BCISE 2 MG/0.85 ML SUBCUTANEOUS AUTO-INJECTOR
GLP-1 RA	00310654004	EXENATIDE MICROSPHERES	BYDUREON BCISE 2 MG/0.85 ML SUBCUTANEOUS AUTO-INJECTOR
GLP-1 RA	00310654085	EXENATIDE MICROSPHERES	BYDUREON BCISE 2 MG/0.85 ML SUBCUTANEOUS AUTO-INJECTOR
GLP-1 RA	00169291115	INSULIN DEGLUDEC/LIRAGLUTIDE	XULTOPHY 100/3.6 100 UNIT-3.6 MG/ML (3 ML) SUBCUTANEOUS INSULIN PEN
GLP-1 RA	00024576105	INSULIN GLARGINE/LIXISENATIDE	SOLIQUA 100/33 100 UNIT-33 MCG/ML SUBCUTANEOUS INSULIN PEN
GLP-1 RA	00169280015	LIRAGLUTIDE	SAXENDA 3 MG/0.5 ML (18 MG/3 ML) SUBCUTANEOUS PEN INJECTOR
GLP-1 RA	00169406012	LIRAGLUTIDE	VICTOZA 2-PAK 0.6 MG/0.1 ML (18 MG/3 ML) SUBCUTANEOUS PEN INJECTOR
GLP-1 RA	00169406013	LIRAGLUTIDE	VICTOZA 3-PAK 0.6 MG/0.1 ML (18 MG/3 ML) SUBCUTANEOUS PEN INJECTOR
GLP-1 RA	00024574502	LIXISENATIDE	ADLYXIN 10 MCG/0.2 ML-20 MCG/0.2 ML SUBCUTANEOUS PEN INJECTOR
GLP-1 RA	00024574702	LIXISENATIDE	ADLYXIN 20 MCG/0.2 ML SUBCUTANEOUS PEN INJECTOR
GLP-1 RA	00169413211	SEMAGLUTIDE	OZEMPIC 0.25 MG OR 0.5 MG (2 MG/1.5 ML) SUBCUTANEOUS PEN INJECTOR
GLP-1 RA	00169413212	SEMAGLUTIDE	OZEMPIC 0.25 MG OR 0.5 MG (2 MG/1.5 ML) SUBCUTANEOUS PEN INJECTOR
GLP-1 RA	00169413602	SEMAGLUTIDE	OZEMPIC 1 MG/DOSE (2 MG/1.5 ML) SUBCUTANEOUS PEN INJECTOR
GLP-1 RA	00169413611	SEMAGLUTIDE	OZEMPIC 1 MG/DOSE (2 MG/1.5 ML) SUBCUTANEOUS PEN INJECTOR
GLP-1 RA	00169431413	SEMAGLUTIDE	RYBELSUS 14 MG TABLET
GLP-1 RA	00169430313	SEMAGLUTIDE	RYBELSUS 3 MG TABLET
GLP-1 RA	00169430713	SEMAGLUTIDE	RYBELSUS 7 MG TABLET

GLP-1 RA, glucagon-like peptide-1 receptor agonist; NDC, National Drug Code.
